# A parametric numerical analysis of femoral stem impaction

**DOI:** 10.1371/journal.pone.0268561

**Published:** 2022-05-20

**Authors:** Nicholas E. Bishop, Phil Wright, Martin Preutenborbeck

**Affiliations:** 1 Hamburg University of Applied Sciences, Faculty of Life Sciences, Hamburg, Germany; 2 Depuy-Synthes, Leeds, United Kingdom; University of Vigo, SPAIN

## Abstract

Press-fitted implants are implanted by impaction to ensure adequate seating, but without overloading the components, the surgeon, or the patient. To understand this interrelationship a uniaxial discretised model of the hammer/introducer/implant/bone/soft-tissues was developed. A parametric analysis of applied energy, component materials and geometry, and interactions between implant and bone and between bone and soft-tissues was performed, with implant seating and component stresses as outcome variables. To reduce the impaction effort (energy) required by the surgeon for implant seating and also reduce stresses in the hardware the following outcomes were observed: Reduce energy per hit with more hits / Increase hammer mass / Decrease introducer mass / Increase implant-bone resistance (eg stem roughness). Hardware stiffness and patient mechanics were found to be less important and soft tissue forces, due to inertial protection by the bone mass, were so low that their damage would be unlikely. This simple model provides a basic understanding of how stress waves travel through the impacted system, and an understanding of their relevance to implantation technique and component design.

## Introduction

The majority of joint replacements worldwide is anchored by press-fit, with numbers increasing [1–3]. Press-fit femoral stem components, and the broaches necessary to prepare the cavity, are impacted into the bone by the surgeon using a hammer and an introducer device. This procedure is well-established, but systems and techniques are variable [4]. Suitable seating of the implant is desired, without damage to the surgeon, implant system or patient. Too little seating can lead to implant loosening and too much can cause periprosthetic fracture of the bone [5, 6]. There is also increasing concern for the health of the surgeon [7–12], who deals with high magnitude repetitive forces in uncomfortable positions. Due to the repetitive nature of impaction, engineers need to understand fatigue in such systems in order to optimise their design [13]. This might involve minimising the energy applied by the surgeon to seat the implant, which might also reduce damage to the rest of the system. The impaction face of orthopaedic hammers becomes dented and splayed with use, and inserter devices are reported to fracture. These observations cause concern to the companies, but they are not publicised. Preclinical testing now involves repeated impaction of implant insertion tools.

Direct relationships can be demonstrated between increased impaction energy (or force) and increased seating for various implants [14–20]. But excessive seating can cause periprosthetic fracture [5, 6]. There is hope that proper implant seating can be predicted by dynamic analyses of vibrations measured during impaction [21–26]. These studies have involved dynamic experimental and analytical work, mainly of acetabular cup and femoral stem seating.

Despite such considerable current interest in the mechanics of orthopaedic impaction there remains little analysis of the load transfer mechanisms from hammer through to soft tissues and there remains little evidence-based information for the surgeon or the designers regarding technique or tools. The influence of the design of the hammer, the implant-introducer components, the stem, as well as the mechanical state of the patient, on implant seating and cyclic stressing of the components is not yet documented.

A logical design aim for the system would be to achieve adequate implant seating, while ensuring surgeon comfort, preventing implantation system breakage, and avoiding damage to patients’ tissues. The purpose of the study was therefore to investigate the effects of impaction technique by the surgeon, implant-system and patient parameters on stresses in the introducer and on implant seating. A simple dynamic model of an impacted femoral stem implantation system was developed to simulate axial load transfer during implantation of orthopaedic implants.

## Materials and methods

**Fig 1:**
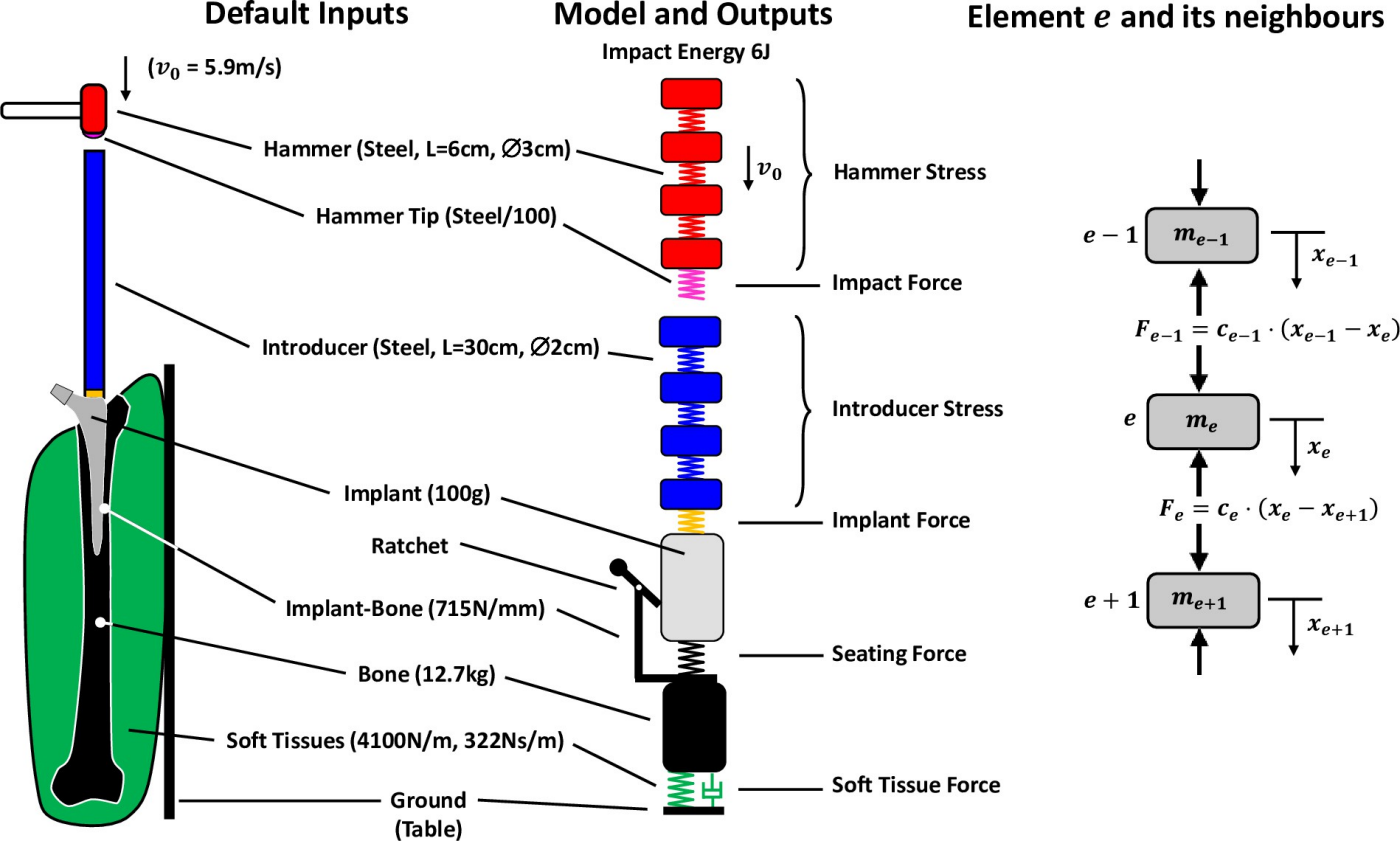
Axisymmetric model of a hammer/tip/introducer/implant/bone/soft-tissues/operating-table with a schematic of the discretised elements (masses, springs, damper) and the forces *F* acting on a mass element *e* due to the deformations *x* of the springs (or damper) on either side. Implant seating friction was simulated by a one-way ratchet between implant and bone, in parallel with a stiffness element (black spring). Nominal values are given, which are listed with details in Fig *2*.

A uni-axial hammer/introducer/implant/bone/soft-tissue model of a femoral implantation system was developed (Fig 1) to determine the dynamic displacement and force distributions in the hammer, introducer, stem, bone and soft tissues. These components were modelled as a series of discrete masses connected with linearly elastic springs, representing linearly elastic materials and interfaces. A single spring element was used to represent the impaction tip of the hammer. Tension and compression forces were permitted throughout the system, apart from at the hammer-introducer impact interface, where only compression forces were permitted, with interface separation otherwise. The interface between the introducer and the stem was permitted to transfer both tension and compression forces because the introducer is generally clamped or screwed to the stem. The stem was considered as a single lumped mass. The combined axial components of the friction resistance and radial press-fit forces during seating of the implant were represented by axial force-displacement data measured experimentally for quasistatic push-in of a porous-coated stem in good quality cadaveric bone. This approach is detailed and validated in [27]. Relative displacement between stem and bone was permitted only in the seating direction, representing irreversible implant seating due to friction. The bone and soft tissues were modelled as a mass-spring-damper component, with experimentally derived parameters [16].

Discrete masses *m*_*e*_ and spring constants *c*_*e*_ of each of *n* elements *e* were calculated for the hammer and introducer according to the density *ρ* and elastic modulus *E* of the components and their assumed length *L* and cross-sectional area *A* (derived from the diameter):

$$m_{e}=\rho \cdot A\cdot L∕n,$$

$$c_{e}=E\cdot A\cdot n∕L.$$

For each element *e*, the sum of the forces acting on each element with mass *m*_*e*_ was related to the acceleration ${\ddot{x}}_{e}$ of each element mass *m*_*e*_ (see Fig 1) according to Newton’s second law of motion:

$$F_{e-1}-F_{e}=m_{e}\cdot {\ddot{x}}_{e}.$$ (1)

The spring force *F* is the product of the spring constant *c* and the spring deformation given by the relative displacements *x* of the elements on either side:

$$F_{e}=c_{e}\cdot (x_{e}-x_{e+1}).$$ (2)

Substituting *Eq 2* in *Eq 1* and rearranging gives the acceleration ${\ddot{x}}_{e}$ of each mass element *e*:

$${\ddot{x}}_{e}=[c_{e-1}/m_{e}\;\left(-c_{e-1}-c_{e}\right)/m_{e}\;c_{e}/m_{e}]\cdot {\left(x_{e-1}\;x_{e}\;x_{e+1}\right)}^{T}.$$ (3)

For the implant-bone interface the spring constant *c*_*e*_ = *c*_*interface*_ was derived from experimentally measured force-displacement implant push-in data ([27], see above). Friction seating of the stem was permitted for:

$${\dot{x}}_{implant}>{\dot{x}}_{bone}.$$

The last element was fixed rigidly at its end (soft tissues to rigid operating table) and a damping component representing the soft tissues was introduced in parallel to the spring, generating a resistance force *F*_*b*_ proportional to the velocity $\dot{x}$ of the bone, where *b* is an experimentally derived damping constant:

$$F_{b}=b\cdot \dot{x}.$$ (4)

The system *(Eq 3)* was integrated numerically over all elements to update the element positions *x* and velocities $\dot{x}$ after time-step Δ*t*. A Backward Euler scheme was employed as a stable method for such problems. Iteration was continued until end-time *T*, which was generally 0.4ms for each hit, to capture implant seating. Each impaction simulation was performed for *T* = 0.4*s* to capture the much longer bone deflection period. Spring forces *F*_*spring*_ were then recovered from the element positions *x* according to *Eq 2*, and axial stresses *σ* in the hammer and introducer were calculated according to the force *F*_*spring*_ in each spring and the cross-sectional area *A*, where

$$\sigma =F_{spring}∕A.$$

The seating force *F*_*seating*_ of the implant in bone was derived from the displacement between implant and bone and the experimentally measured implant-bone interface resistance *c*_*interface*_ (described above), according to:

$$F_{seating}=c_{interface}\cdot (x_{bone}-x_{implant}).$$

Soft tissue damper forces *F*_*b*_ were recovered according to *Eq 4*.

**Fig 2:**
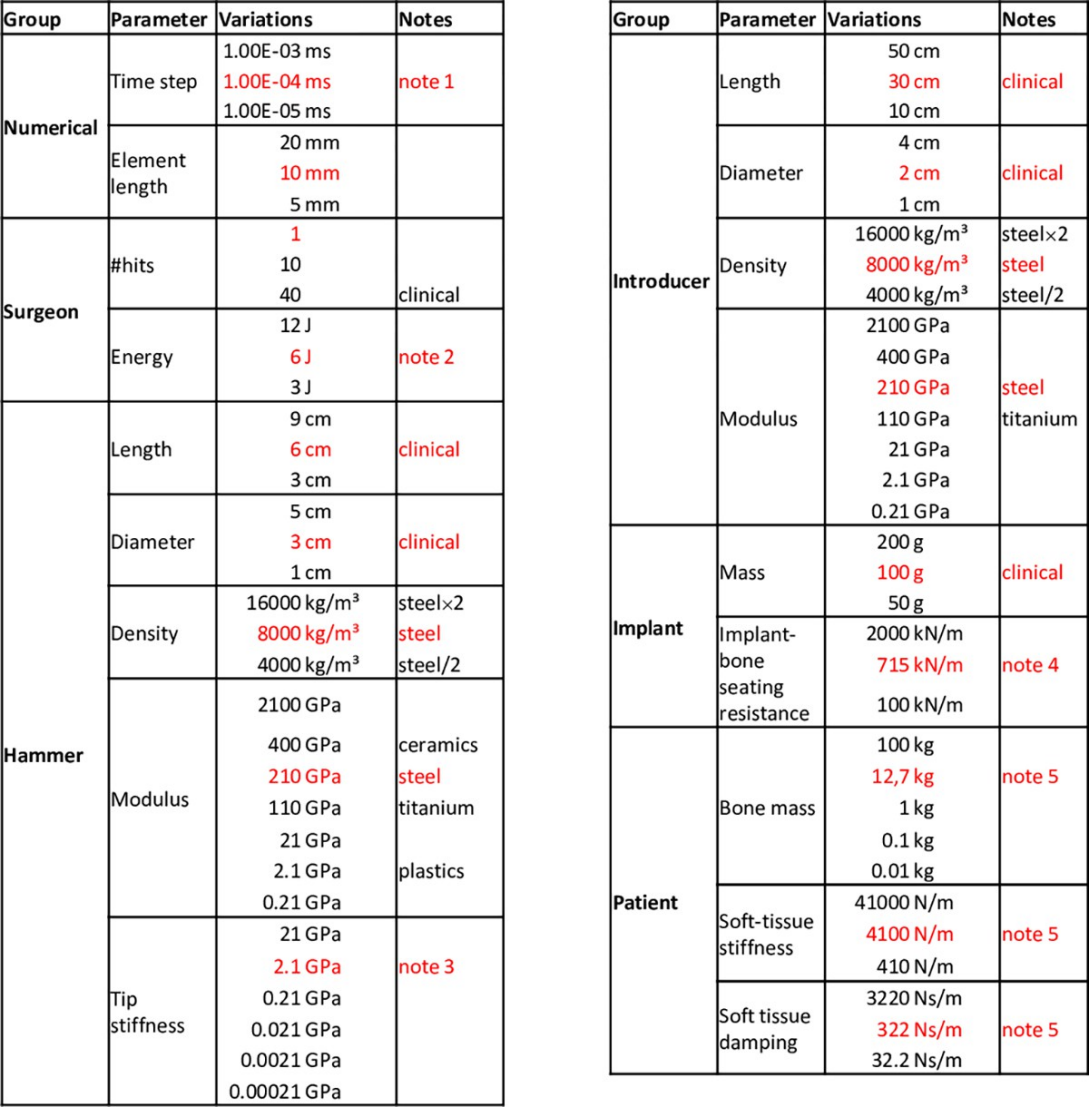
Nominal values for each parameter (red) and their variations. Note1: This value results in 1000 points representing the nominal applied force-time impaction impulse; Note2 from [17]; Note3: This value results in a clinically measured impaction force [16]. Videos of the load transfer for the varied hammer-introducer stiffness (nominal, +/-order of magnitude) can be found as S1–S3 Files; Note4: Data for porous-coated stem in cadaveric femur [27]; Note5: from [16].

Initial values for all parameters are given in Fig 2, according to realistic and published magnitudes (sources noted in the caption). Individual variations were made to these values to assess sensitivity. Note that the hammer velocity *v*_0_ at the onset of impact was varied to ensure constant applied kinetic energy *E* for a given hammer mass *m*, according to $E=\frac{1}{2}\cdot m\cdot {v_{0}}^{2}$.

**Implant seating force** and **maximum introducer stresses** were considered as “**output”** variables and their variation in terms of sensitivities to the parametric variations in “**input”** variables were determined. Sensitivities were calculated as the slope of the linear best-fit to the change in % **output** plotted against the change in % **input** from the nominal values for each parameter given in Fig 2. Convergence was tested similarly.

## Results

### Convergence

Sensitivities of the study output variables “implant seating force” and “introducer stress” to time-step and element-length were very low (<3.5%), indicating temporal and spatial convergence of the model.

### Load transfer (nominal parameter magnitudes)

**Fig 3:**
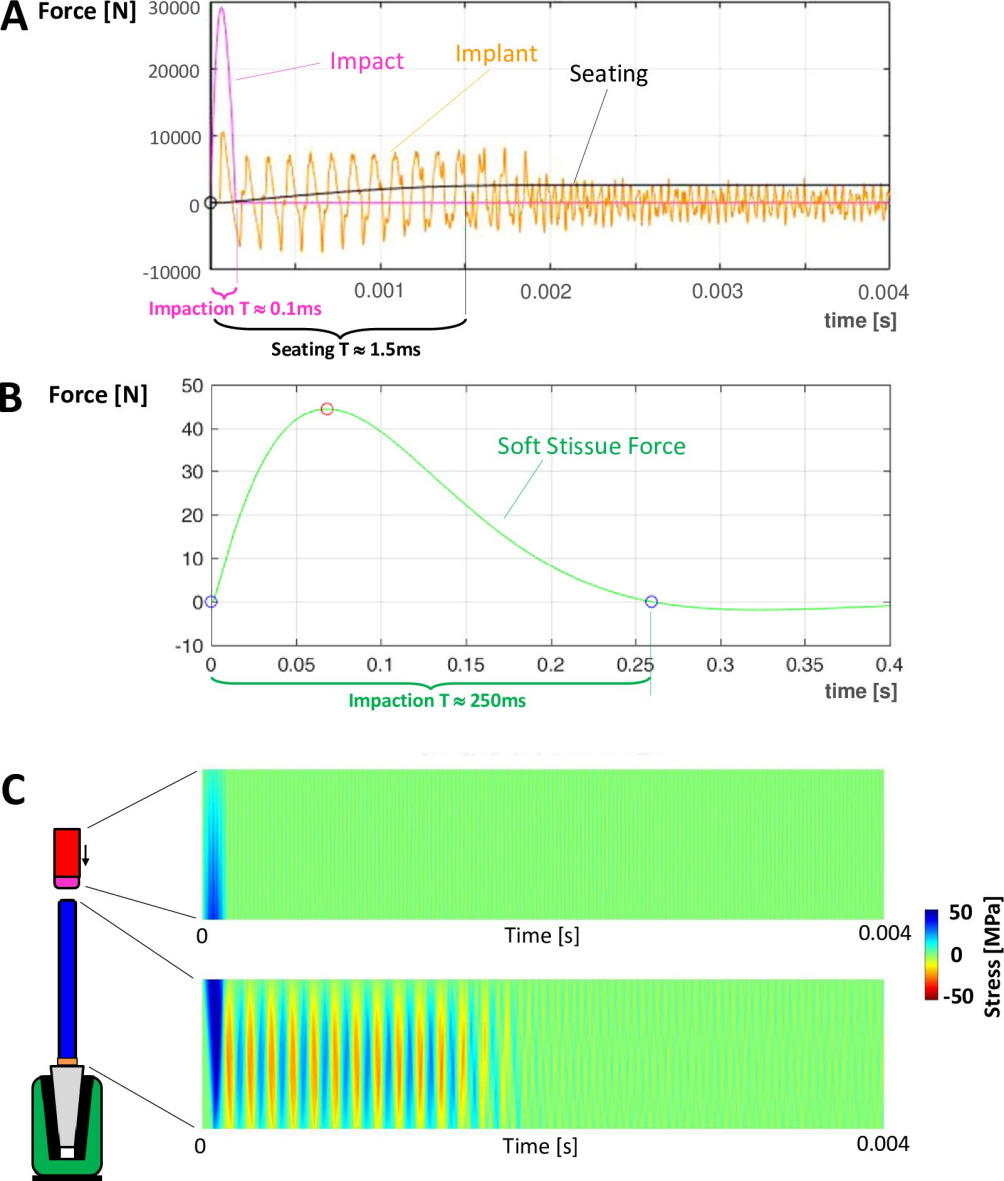
Force outputs are shown over time for the nominal variable magnitudes. Positive forces are compressive, negative tensile. **A**: Contact force between hammer tip and introducer (magenta, period 0.1ms), force between introducer and implant (orange), seating force of implant in bone (black, period 1.5ms). A period of 4ms is shown. **B**: Soft tissue forces (green). The compression period is 250ms. Note that the period shown is 0.4s (100 times greater than the graph above). **C:** Stress distributions along the lengths (vertical) of the hammer (above) and introducer (below) over the same 0.004s time period as for plot A (horizontal). The load transfer can be observed dynamically in S1 File.

For the nominal parameter magnitudes, the impact force between hammer and introducer (Fig 3A) had a peak of ~30kN and a period of ~0.1ms (magenta line), similar to clinical magnitudes. This led to an oscillating force between introducer and implant (orange line) with an amplitude of ¼—⅓ of the peak applied force and a similar period (~0.1ms, 8kHz). Implant seating (black line) occurred during ~10 force reversals between introducer and implant and took ~10 times longer than the hammer impaction period. The magnitude of the seating force was ~10% of the applied force and ~30% of the force acting between introducer and implant. Once the stem had seated, the force between introducer and implant reduced by ~50% and the vibration frequency increased (22kHz). A single deflection of the bone (Fig 3B) was observed with a soft tissue force of 3 orders of magnitude less than the impaction force (~44N) and with a period 3 orders of magnitude greater (~0.25s).

(Fig 3C and S1 File) shows the stress distributions through the hammer and introducer during implantation for the nominal parameter values and seating lasted ~2ms. Maximum stress in the hammer acted in compression at its contact tip during impaction. Maximum stresses in the introducer were compressive and occurred during the hammer strike. This impaction stress wave moved distally along the introducer (at the speed of sound in the introducer ~6000m/s, which is observed by a slight diagonal shape to the right from proximal to distal for the blue compression stress plot). It has a similar magnitude to the impaction stress but with a shorter period, and is reflected at each end of the introducer. Following seating of the implant (~2ms), vibrations perpetuated in the introducer and hammer, but with much smaller amplitudes and higher frequencies (hammer 42kHz, introducer 22kHz).

### Sensitivities

**Fig 4:**
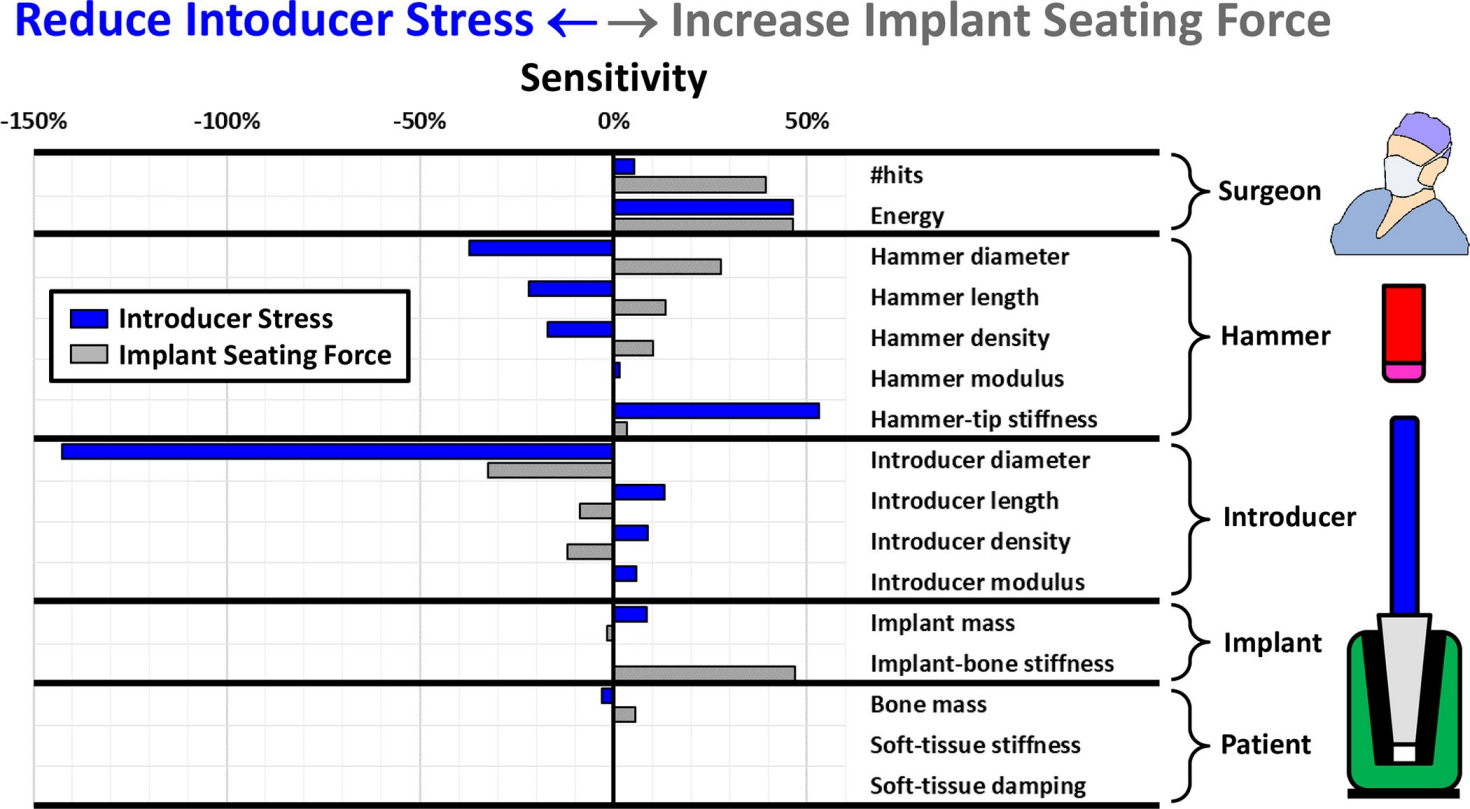
Sensitivities of introducer stress and implant seating force to each parameter variation are shown. Sensitivity is the slope of the best fit through a plot of percentage change in output vs percentage change in input, relative to nominal values. The aim of the study is to find parameters that will decrease stresses (blue with negative sensitivity) and increase implant seating force (grey).

Fig 4 shows sensitivities of introducer stresses and seating forces to parametric variations. For example, -50% would imply that increasing the input variable by 100% from the nominal would decrease the output to 50% of the nominal value. With the aim of decreasing introducer stresses (blue bars), and increasing implant seating (grey bars) blue bars should be negative and grey bars should be positive. Reducing the applied energy and number of hits would reduce stresses but would also decrease seating (“surgeon” parameters). However, stresses decreased with increasing hammer diameter, length and density, which are all directly related to increasing hammer mass (“Hammer” parameters). The opposite is observed for introducer length and density (“Introducer” parameters), suggesting introducer mass should be decreased. However, reducing the mass by decreasing the introducer diameter increases stresses, due to the reduced cross sectional area. Increased implant-bone implantation resistance (e.g. stem roughness) increased the seating force without increasing stresses in the introducer, perhaps allowing less energy to be applied (“Implant” parameters). Introducer stresses can be decreased by decreasing the implant mass and increasing the effective bone mass (“Implant” parameters). Sensitivities of implant seating and introducer stresses to material stiffnesses of the hammer and introducer are rather low. Increasing hammer tip stiffness seems to increase introducer stresses and might better be avoided. These sensitivities are presented below in greater detail, with some analysis of their interactions.

### Hammer and introducer

**Fig 5:**
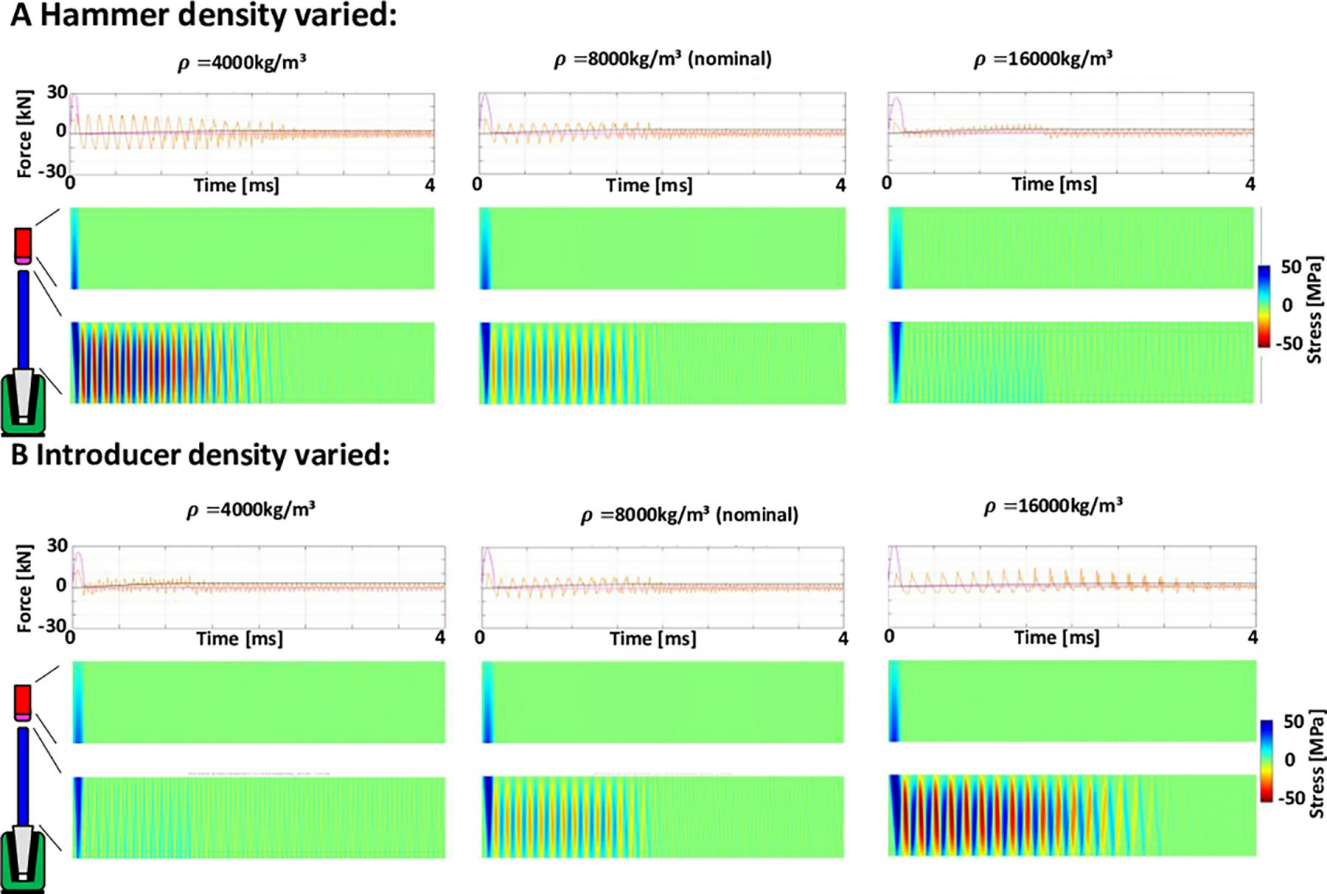
Note: Same colour-coding as Fig 3. **A & B** graphs: Force outputs are shown over a time period of 4ms. Positive forces are compressive, negative tensile. Contact force between hammer tip and introducer (magenta), force between introducer and implant (orange), seating force of implant in bone (black). **A & B** contour plots: Stresses along the length of the hammer (Upper) and introducer (Lower) over time.

Sensitivity results suggested that parameters increasing hammer mass and decreasing the introducer mass fulfilled the aims of increasing seating and decreasing introducer stresses (Fig 3). Effects of hammer density on load transfer are shown in Fig 5A and are representative of the effects of the other variables related to mass. Increasing hammer density decreases the peak applied force because the hammer velocity is decreased to maintain constant kinetic energy of impaction. This reduces force peaks and stresses in the introducer. However, the impact period is increased, and this seems to increase seating and increase the rate of seating somewhat.

The results for the introducer are the opposite of those for the hammer, as can be seen by the increase in peak applied force and period for increasing introducer density (Fig 5B), which increases stresses in the introducer but decreases seating, which also takes longer, due to the lower frequency of the vibrations. Similar effects were observed for increasing introducer length.

### Hammer tip stiffness and surgeon parameters

**Fig 6:**
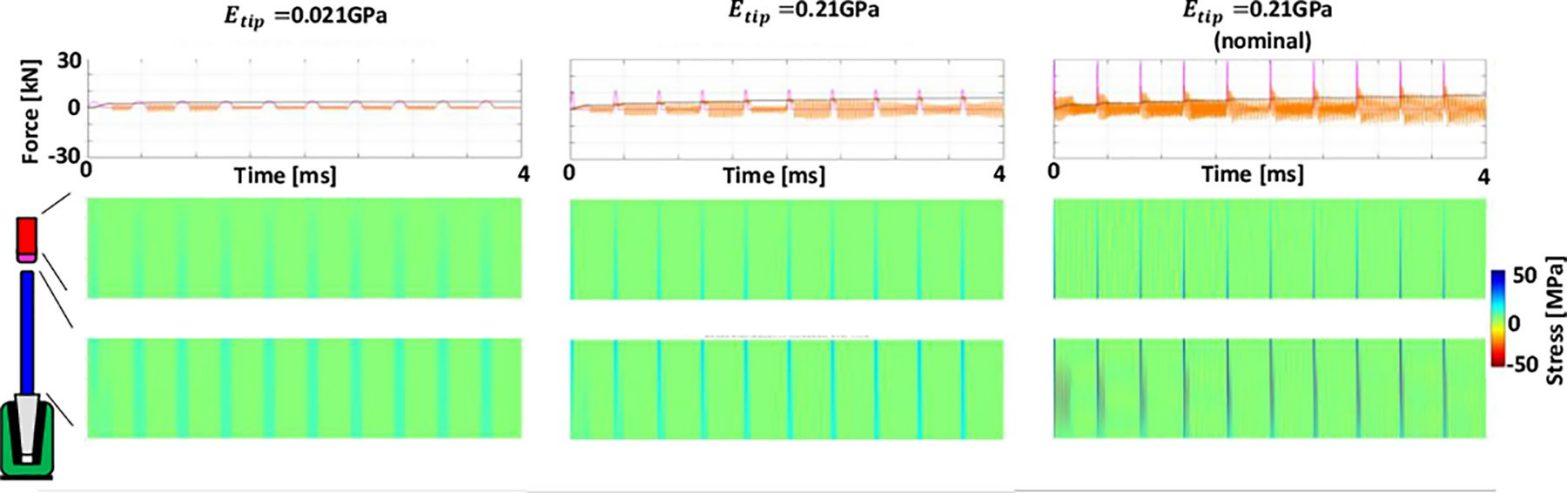
Note: Same colour-coding as Fig 3. Upper: Forces and stresses are shown for 10 consecutive hits, with hammer tip stiffness increasing from left to right. The right-hand plots (*E*_*tip*_ = 2.1GPa) are for nominal parameter magnitudes. Decreasing the tip stiffness towards the left decreases the peak impaction force and increases its period. This reduces the seating force and for the softest tip seating becomes non-progressive for consecutive impactions. Forces between the introducer and the implant become a smaller proportion of the impaction force and stresses in the introducer decrease. Note that these three cases can be visualised dynamically for the first hit in S1 File (above middle, nominal), S2 File (above left, 0.1xnominal), S3 File (above right, 10×nominal).

Increasing the hammer tip stiffness, applied energy and number of hits (separately) increased introducer stresses, as well as implant seating (Fig 6 and S1–S3 Files). These outcomes tend to be related to increased peak applied forces. Peak impact forces increase with hammer tip stiffness, while their period decreases (Fig 6, magenta lines). This results in greater implant seating forces (which also increase with consecutive impaction), but also to greater stresses in the introducer (compare varied hammer tip stiffness in S2 & S3 Files with nominal clinical tip stiffness in S1 File to observe dynamically the more distinct and shorter-wavelength reflected waves for increasing tip stiffness).

**Fig 7:**
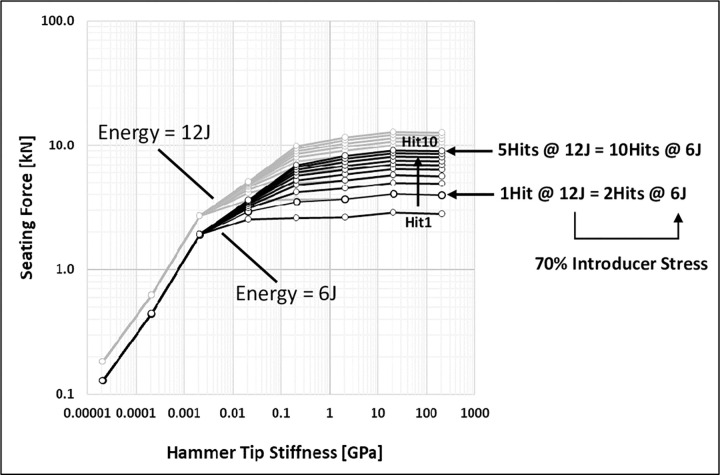
The implant seating force is the anchorage force achieved between implant and bone. It decreases with decreasing hammer tip stiffness and seating becomes less progressive with consecutive impactions (10 hits shown). Clinically measured impaction forces are achieved for a hammer tip stiffness of ~2GPa. Increasing the applied energy increases the seating for all hammer tip stiffnesses by the same proportion.

This seating behaviour is summarised in Fig 7 for a larger range of hammer tip stiffnesses and two applied energy levels. For tip stiffnesses greater than 0.021GPa, consecutive impactions led to progressive seating. The plot suggests an interaction between applied energy and number of hits. These parameters are the responsibility of the surgeon. Doubling the energy increased seating by the same proportion for all hammer tip stiffnesses (constant vertical shift on the log scale Fig 7). Thus, less energy can be applied with more hits to achieve the same seating. This reduces introducer stresses by 30% in this example.

### Introducer stresses

**Fig 8:**
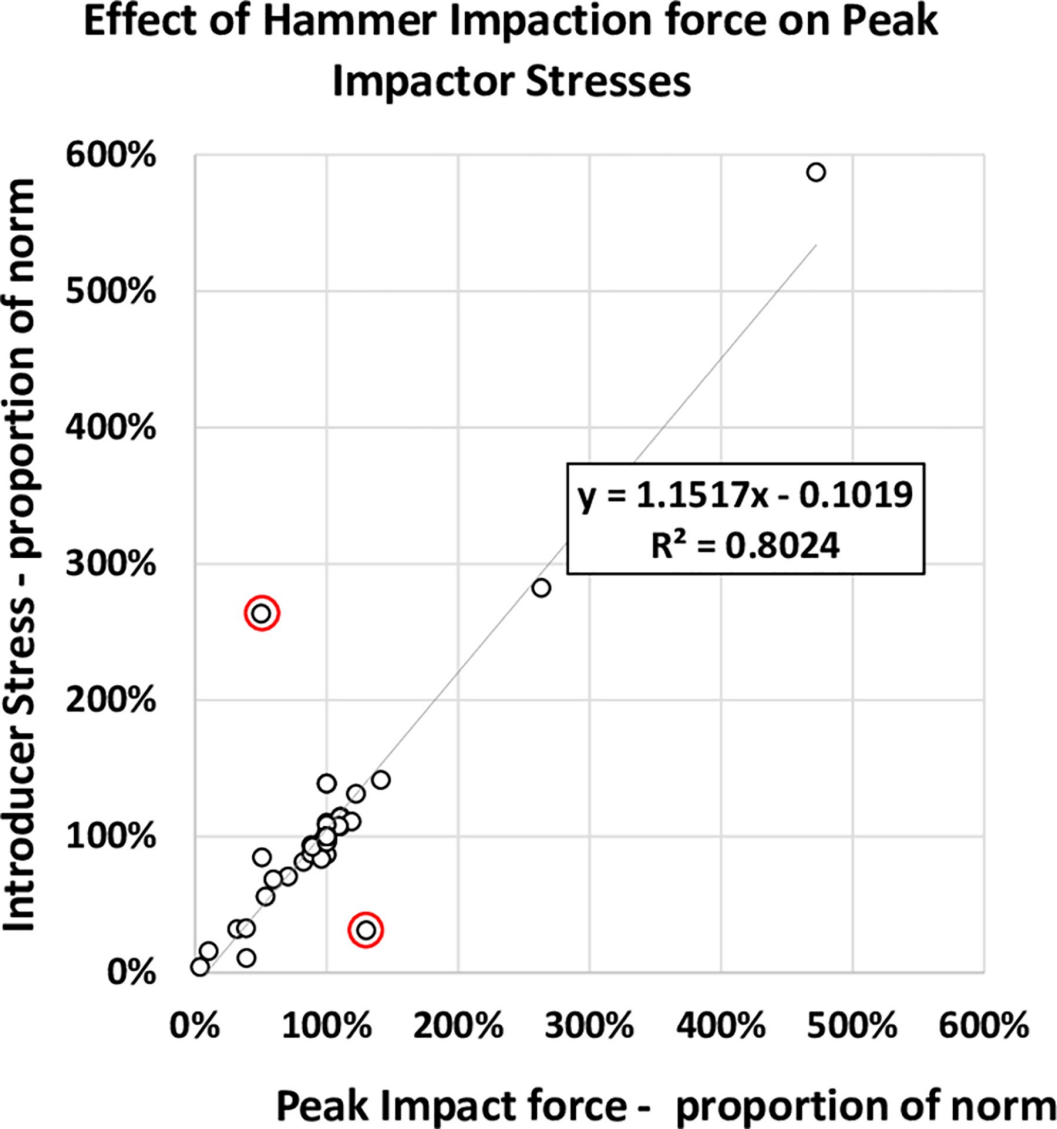
Introducer stresses are plotted against the peak impact forces for all parametric variations tested and are mostly found to be proportional. Values are scaled to those for the nominal model parameters. The outliers (circled in red) are for changes in diameter of the introducer for which stresses are inversely proportional to the square of the diameter (cross sectional area).

Introducer stresses appear to be related to the magnitude of the peak applied force. This relationship is plotted for all variations made in this study (Fig 8). The main outliers are the two for variations in the diameter of the introducer, which has an overriding effect on stresses by changing the cross-sectional area. There was a no clear correlation between introducer stress and implant seating force, which are the two output variables (not shown).

### Implant parameters

**Fig 9:**
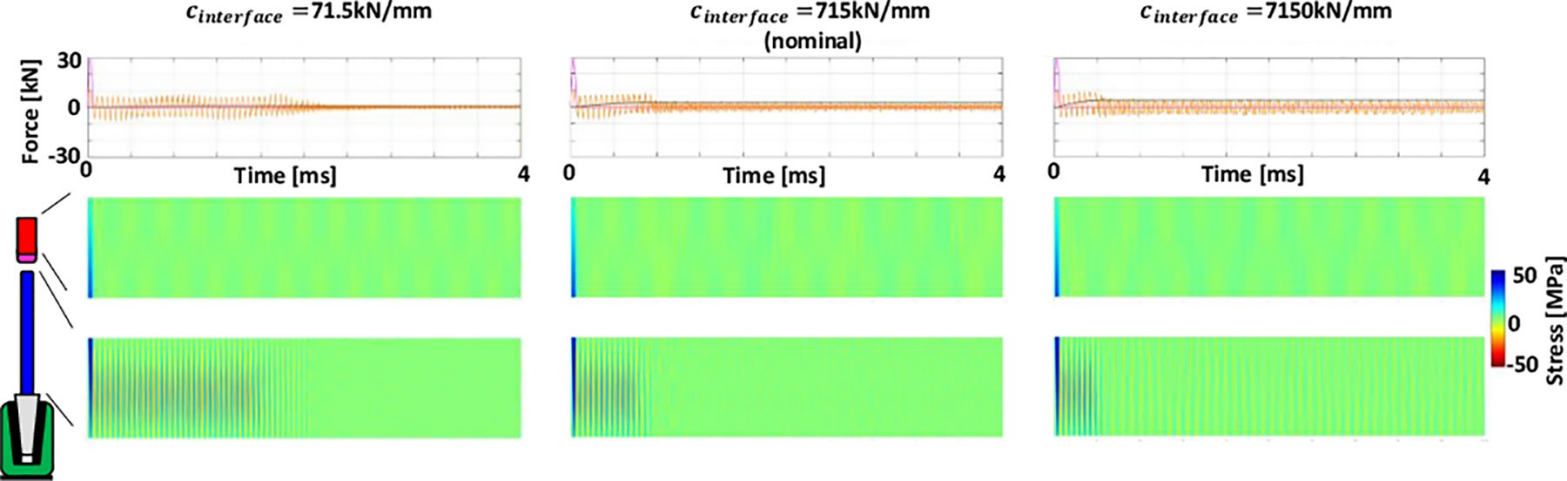
Note: Same colour-coding as Fig 3. Increasing the implant seating resistance of the implant into the bone has no effect on the impaction force (magenta) or the magnitude of the force between introducer and implant (orange). However, implant seating force (black) increases and is more rapid, with fewer stress oscillations in the introducer (contour plots).

Increasing the resistance to stem insertion into the bone (rougher implant surface, better bone quality) increases the implant seating force (anchorage force), without influencing the introducer stress magnitudes during seating (Fig 9. Note: Same colour-coding as Fig 3) because impaction force magnitudes are unaffected. The rate at which seating occurs increases, with less oscillations before seating. Seating and introducer stresses are rather insensitive to implant mass.

### Patient parameters

**Fig 10:**
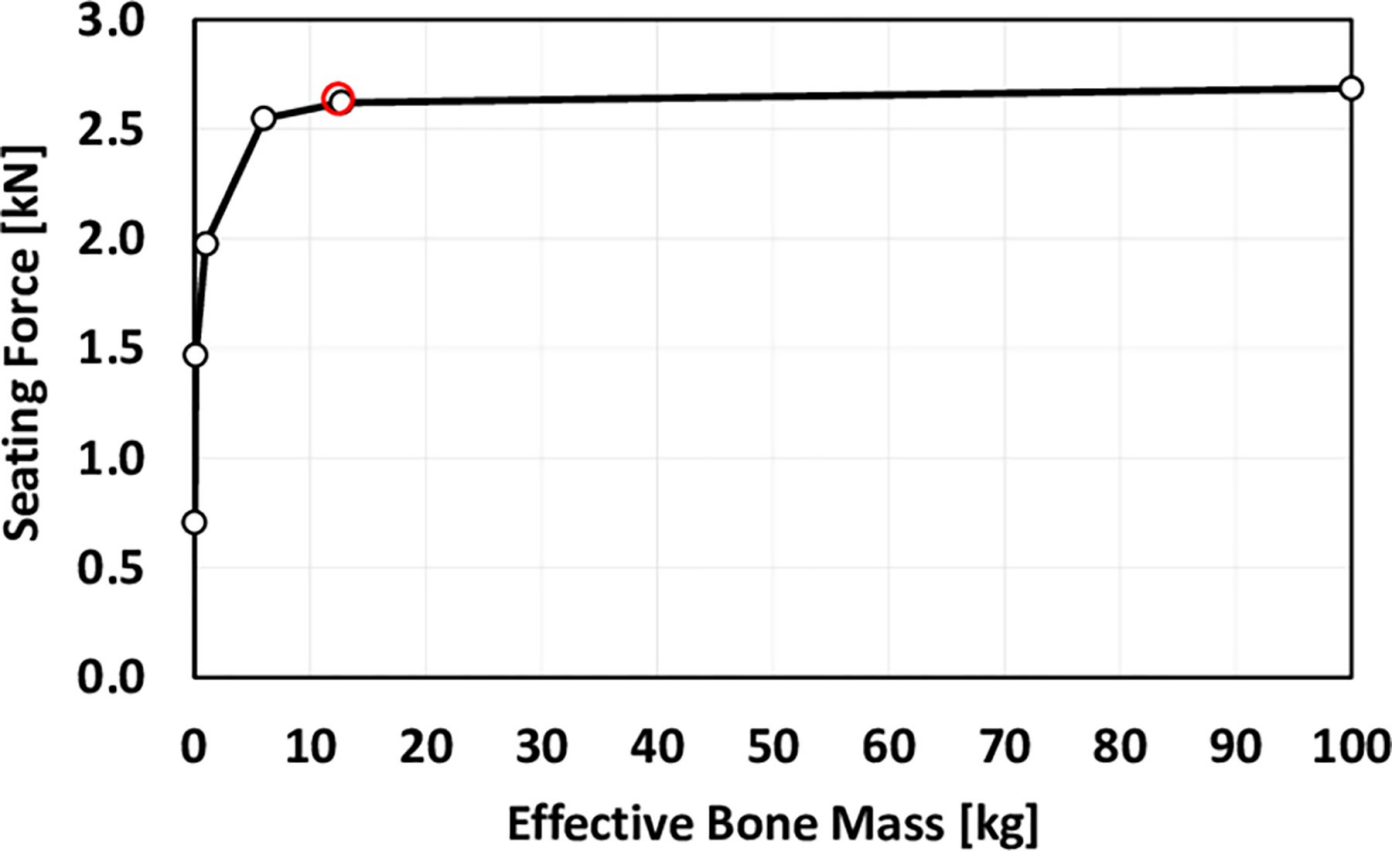
The implant seating force is plotted for the effective bone mass. The nominal condition tested is shown circled, according to published data [16] and results in a maximum seating force. This decreases for effective bone masses of less than the nominal value.

Implant seating and Introducer stresses were rather insensitive to patient tissue parameters. It is noted that impaction and implant seating are completed long before significant motion of the bone occurs (Fig 3C), so that the bone effectively acts as a rigid boundary during implant seating. However, further analysis (Fig 10) suggests that for an effective bone mass of less than 6kg seating reduces dramatically because the inertia of the bone is no longer great enough for it to act as a rigid base during implant seating. In this case, soft tissue parameters would start to affect bone deflection and implant seating. Soft tissue forces were relatively low (<50N) compared to body weight.

## Discussion

In this study, seating of a press-fit femoral stem by impaction was modelled. Dynamic seating of orthopaedic implants has been documented experimentally and related to hammer forces [14–20]. Generally, greater hammer energy is observed to increase implant stability [28–30], which is also reflected by the current model, but can also increase the risk of periprosthetic fracture [17]. Great efforts have also been made to develop analyses of hammer force [21, 22, 26], implant acceleration [23] and acoustic signals [24, 25] to detect implant seating and to provide intraoperative feed-back to the surgeon, thus maximising stability while limiting potential bone damage. However, such studies do not tend to assess the dependency of implant seating on the factors of interest in the current study, related to surgeon, tools, implant and patient. This limits the comparisons that can be made. Signal analyses of vibrations observed for the current model would provide limited comparison with published vibration-based studies, and is beyond the scope of our study. This is discussed in the “Model Limitations” section, as a limitation to the validation of the current model. However, in the same section a comparison with experimental data is presented, which provides support for the model.

The aim of this study was to find parameters that might reduce the surgeon effort necessary to seat the implant and prevent the risk of hardware failure. Consideration of an absolute optimal implant seating position is not considered in this sensitivity study and is left to the experts cited above.

### Load transfer

The linearly elastic mass-spring model presented is presumed to provide a suitable representation of dynamic load transfer in typically metal components, which are linearly elastic continua, with very little material damping [31]. The analysis showed that for realistic parameter magnitudes a single hammer impaction generates a compressive stress wave that travels at the speed of sound through the introducer and reflects against the end of the implant as a tension wave, moving back up the introducer, and reflecting back down again as a compression wave (Fig 3 and S1–S3 Files). This cycle occurs a number of times during seating, stressing the whole length of the introducer rather evenly in reversing tension and compression and generating seating of the implant due to each compression wave acting between the introducer and the implant. It is noted that distinct stress wave pulses will only be observed if the period (wavelength) of the applied impaction force wave is less than the period the wave takes to travel the length of the introducer. A softer hammer tip increases the impaction period (and decreases the peak force) and will increase the wavelength. Implant seating is progressive during a number of stress reflections in the introducer rod, occurring subsequent to the hammer strike. Some of the impaction energy is lost as frictional work between implant and bone during implant seating but some energy remains in the elastic system after implant seating in the form of higher frequency, lower amplitude vibrations in the hammer and introducer. No material damping was modelled, apart from that of the soft tissues, which attain maximum deflection motion much later (~0.1s) than the implant seating event (~0.001s) and therefore would not damp system vibrations during the seating period. In reality, further energy would leave the system due to material damping and handling by the surgeon and vibrations would die away more rapidly. The initial compressive impaction stress in the introducer was greater in magnitude than the subsequent reflections in the introducer during seating, followed by much smaller amplitude vibrations. Which of these reversing stress cycles could be large enough to propagate fatigue cracks is speculative but may not be limited to the first impaction peak [13].

### Component design

In general, stresses in the introducer were related directly to peak impaction forces (Fig 8). Peak impaction forces and stresses were reduced: **(i)** for parameters increasing the hammer mass (note that for a constant applied energy by the surgeon the higher hammer mass was compensated for by a lower strike velocity); **(ii)** for parameters decreasing introducer mass; **(iii)** for lower hammer tip stiffness. Decreasing the peak applied force, in order to decrease introducer stresses could have the undesired effect of also decreasing implant seating. However, seating is also dependent on factors such as the period of the applied force and the rate at which the force is transferred to the implant. Thus,

- increasing the hammer mass decreases the peak force but increases the period of the impaction force, which has the net effect of increasing implant seating,
- decreasing the introducer density decreases the peak forces but also increases the wave speed, which appears to increase implant seating,
- decreasing the hammer tip stiffness decreases the peak force in the introducer and this also tends to decrease implant seating.

A high hammer tip stiffness may therefore be necessary **(point iii)**, but with a low-density introducer **(point ii)** and perhaps a higher mass hammer **(point i)**, although this may increase discomfort to the surgeon. It is noted that the hammer tip stiffness increases with tip material stiffnesses at the impaction interface, and with increasing radius or contact area of the interface surfaces. These things could be achieved by powered impaction tools [32, 33] with a predefined impaction interface geometry. The maximum effective tip stiffness tends to the magnitude of the lower of the stiffness of the bulk hammer or introducer materials. The nominal effective hammer tip stiffness of 2.1GPa was chosen in this study to result in impaction forces matching experimentally measured magnitudes of ~30kN [34] (see Fig 7) and seating was predicted to be progressive with consecutive impactions (Fig 6, implant seating: black lines), as observed clinically.

### Surgeon parameters

Fig 7 demonstrates that it may be advantageous to achieve desired implant seating force by decreasing the applied energy so as to reduce hardware stresses and surgeon effort, while applying a greater number of hits. This possibility will require further investigation, with regard to the relative effects on fatigue failure and surgeon comfort. It should be noted that the assumption of constant energy consecutive impactions presented in this study does not reflect the clinical situation, in which the surgeon tends to increase the impaction energy with consecutive impactions. Increasing energy will increase introducer stresses and more constant energy impaction may be preferable, for example, using powered impaction tools [32, 33].

### Implant parameters

The mass of the implant had little effect on stresses or seating, probably because its mass represents only a small proportion of the accelerated mass together with the introducer (~13% for nominal values used above). The model suggested that seating force could be increased by increasing the implant seating resistance (rougher implant [35] or stiffer bone). This also decreases the number of stress cycles in the introducer during seating, without increasing the stress magnitudes. This could reduce fatigue.

### Patient parameters

Implant seating and introducer stressing were found to be rather insensitive to the effective mass of the bone for magnitudes estimated [16] for clinical impaction. In this range the bone acts like a rigid base and deflects with a much longer time period than the implant seating period (Fig 3). For this bone mass, the stiffness and damping of the soft tissues had no effect on the implant seating mechanics, and forces in the soft tissues (<50N) would seem to be too low to cause any tissue damage. Minimal effects of the patient mechanics on implant seating have been noted in other studies [14, 15]. However, since the available data is limited (the nominal bone mass of 12.7kg was derived from implantation in a single 60 kg patient [16]) the possibility of a much lower effective patient mass should be acknowledged, with the consequence of reduced implant seating (Fig 10), as the bone then moves with the implant, and of increased soft tissue stressing.

### Model limitations

The current model was constructed to relate impaction parameters, design of the implant and implantation system, and patient parameters to implant seating and system stressing. Ideally, such a model should be validated against measurements made intraoperatively of all the input and output parameters investigated. Such data is unavailable! Implant seating in bone can be measured quite easily, using non-invasive systems [33, 36]. Stressing can be derived from surface strain measurements. Implant seating measurements have been published [33, 36], but are not generally related to the input variables of interest in the current study. Many studies of impaction dynamics have been published, often addressing the prediction of sufficient and safe implant seating, by signal processing of impaction accelerations [23], forces [21, 22, 26] or acoustic measurements [24, 25]. Use of such data to validate the current model would require complex signal processing, which is not within the scope of the study and would be of questionable value.

The authors have recently published experimental measurements of implant seating by impaction of a fluted titanium alloy revision stem in foam surrogates for bone [27]. A single lumped mass model of implant seating based on quasistatic measurements of resistance to implantation was validated against the data. This provides support for the validity of the distributed mass model presented in the current study, which incorporates the same implant seating algorithm. Indeed, the current model matches implant seating magnitudes for the single lumped mass model well (mean 1% difference over 10 hits, maximum deviation of 17% for the first hit, see S5 File), despite a constant 50kN load being applied by the hammer in the current model and the more variable measured forces being applied to the published model. A ~4.5kN implant seating (anchorage) force simulated by the models, was achieved after 10 impactions, which related to ~34mm implant seating displacement. The measured seating force after 10 hammer hits was 3.4kN, which was overestimated by the models by one third (see comparison graph in S5 File). This overestimation was considered to be due to higher seating resistance experimentally under impaction, which could not be accounted for by the quasistatic resistance measurements used as input to the model. The implant seating period was also noted to be similar to experimental measurements (~0.01s).

The current model can therefore predict realistic seating under multiple impactions. This implies that axial load transfer is modelled correctly, and it could therefore be deduced that axial stresses are also correctly modelled, despite a lack of experimental strain data, which could allow direct validation. Consequently, axial vibration frequencies should also be correctly modelled in the current model and indeed, the impaction stress waves were observed to travel along components at the speed of sound *c*, based on the elastic modulus *E* and density of the material *ρ* according to $c=\sqrt{E/\rho }$ (for example, *c*_*steel*_≈6000*m*/*s*), as would be expected. It is noted that the dependence of implant seating on the hammer tip stiffness (which determines the peak applied force) shown for a wide range in Fig 7 is shown in [27] to be a characteristic saturation function. It was shown that this function can be scaled to any system, according to the peak of the applied force, the applied hammer impulse, the resistance to implant seating and the implanted mass. These similarities provide further confidence that the current model functions correctly over a wide range of impaction conditions.

Realistically, load transfer would involve bending, since many introducer designs have a non-axisymmetric design, and the surgeon does not strike axially [37]. This will introduce shear/bending vibration modes, which will increase stresses and also result in lower fundamental vibration frequencies. Nevertheless, the uni-axial load transfer presented here is informative, as it represents the fundamental axial mode of load transfer, which is responsible for (axial) implant seating, regardless of other superimposed deformation modes. Axial waves were observed to reflect in the introducer a number of times during seating, probably before any resonant bending modes could develop. Of course, more complex models will be necessary to study load transfer in multiple degrees of freedom and detailed geometry will allow local stress concentrations and detailed failure modes of impaction devices to be studied. The current uni-axial model may be particularly relevant to contemporary linear implant insertion systems, and particularly those which employ an axially mounted impaction gun, offering optimal impaction [32, 33].

The implant seating model employed was based on quasistatically-measured force-displacement implant seating resistance. It is assumed that this can represent the seating resistance for dynamic (impacted) implant seating. This has been confirmed for polyurethane foam bone substitutes [27] but remains to be tested for bone. However, this is a practical method of simulating press fit implicitly. Models presented in other studies include a friction interface between implant and bone [21, 27, 30], but dynamic friction coefficients are difficult to measure, and such contact analyses are complex.

## Conclusion

The dynamic load transfer from surgeon through to soft tissues has not been studied systematically in terms of practical outcomes. The aim of the current study was to identify parameters that influence implant seating and stresses in the introducer tool, and also those that do not. This model might be applied to any linear press-fit system, given experimental implant push-in data to model the press-fit implantation resistance. Indeed, the general findings should also apply to such similar systems as acetabular cups, endoprosthetic broaches and press-fit knee implants. The following generalisations can be made from the simple model presented, but would need to be confirmed in more detailed models and experiments.

1. Reducing the impaction energy will reduce the effort necessary for the surgeon and is desirable. It also decreases stresses in the introducer tool. Reducing the amount of impaction energy necessary to achieve a given seating force can be achieved by:
2. Decreasing the introducer mass,
3. Increasing the seating resistance of the implant in the bone (e.g. stem roughness),
4. A greater number of lower energy hits (with a stiff impaction interface),
5. Increasing hammer mass.
6. Also:
7. Patient condition and implant mass have less effect,
8. Hammer and introducer stiffness have less effect.

## Supporting information

S1 File Hammer tip with nominal stiffness. Shows dynamic force transfer through hammer/introducer/implant/bone/soft-tissues for impaction force representing clinical. Compare with S2 & S3 Files (hammer tip stiffness varied).
(MP4)

S2 File Hammer tip stiffness reduced from nominal. Shows dynamic force transfer through hammer/introducer/implant/bone/soft-tissues for impaction force lower and longer than clinical. Compare with S1 File (nominal hammer tip stiffness).
(MP4)

S3 File Hammer tip stiffness increased from nominal. Shows dynamic force transfer through hammer/introducer/implant/bone/soft-tissues for impaction force higher and shorter than clinical. Compare with S1 File (nominal hammer tip stiffness).
(MP4)

S4 File Parametric implant impaction study data. In the first Sheet each combination of input variables is given in rows with parameters defined in the column headings. Each of the other sheets lists data for an output variable related to the input set in the same row number.
(XLSX)

S5 File Validation of the model. This is a commented plot of implant seating for the current model compared with published data for experimentally measured seating and predictions using a simple model with the measured impaction forces applied.
(DOCX)
