# Supplementary material for: A parametric numerical analysis of femoral stem impaction
Source: PLoS One. 2022 May 20;17(5):e0268561. doi: 10.1371/journal.pone.0268561 (PMC9122192; doi:10.1371/journal.pone.0268561)
Supplement: S5 File — This is a commented plot of implant seating for the current model compared with published data for experimentally measured seating and predictions using a simple model with the measured impaction forces applied. (DOCX) [file pone.0268561.s005.docx]

S5 Supporting Material: Model validation


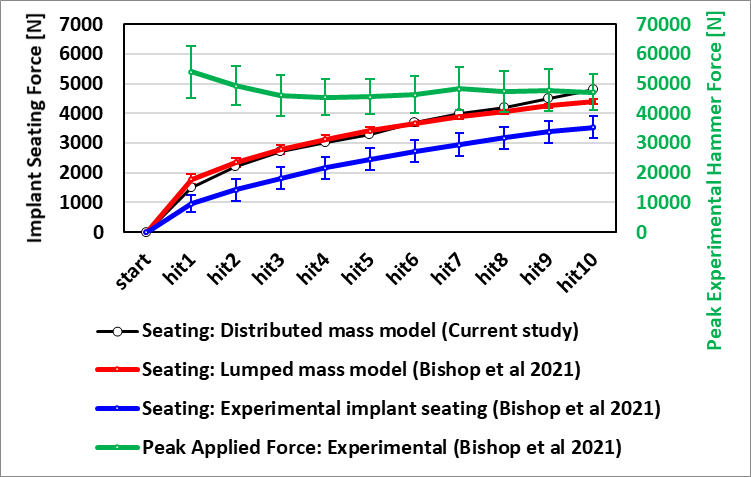


The authors have recently published experimental measurements of implant seating by impaction of a fluted titanium alloy revision stem in foam surrogates for bone (Bishop et al 2021 “A model of implant seating by impaction” Med Eng Phys (97) 47-55 <https://doi.org/10.1016/j.medengphy.2021.09.009>). A single lumped mass model of implant seating based on quasistatic measurements of resistance to implantation was validated against the data. This provides support for the validity of the distributed mass model presented in the current study, which incorporates the same seating algorithm. Indeed, the current model (Black line) matches seating magnitudes for the single lumped mass model (Red line) well (mean 1% difference over 10 hits, maximum deviation of 17% for the first hit), despite a constant 50kN load being applied by the hammer in the current model, while the actual measured forces (Green line: right-hand force scale) were applied to the published model. A ~4.5kN implant seating (anchorage) force simulated by the models, was achieved after 10 impactions (which related to ~34mm seating displacement). The measured seating force (Blue line) after 10 hammer hits was 3.4kN, which was overestimated by the models by one third. This overestimation was considered to be due to higher seating resistance experimentally under impaction, which could not be accounted for by the quasistatic resistance measurements used as input to the model.
